# Testicular Structure and Spermatogenesis in the Naked Mole-Rat Is Unique (Degenerate) and Atypical Compared to Other Mammals

**DOI:** 10.3389/fcell.2019.00234

**Published:** 2019-10-16

**Authors:** Gerhard van der Horst, Sanet H. Kotzé, M. Justin O’Riain, Liana Maree

**Affiliations:** ^1^Department of Medical Biosciences, University of the Western Cape, Bellville, South Africa; ^2^Division of Clinical Anatomy, Department of Biomedical Sciences, Stellenbosch University, Stellenbosch, South Africa; ^3^Institute for Communities and Wildlife in Africa, Department of Biological Sciences, University of Cape Town, Cape Town, South Africa

**Keywords:** spermatogenesis, spermiogenesis, spermiation, staging, Leydig cells, Sertoli cells

## Abstract

The naked mole-rat (NMR) queen controls reproduction in her eusocial colony by usually selecting one male for reproduction and suppressing gametogenesis in all other males and females. Simplified, polymorphic and slow-swimming spermatozoa in the NMR seem to have been shaped by a low risk of sperm competition. We hypothesize that this unique mammalian social organization has had a dramatic influence on testicular features and spermatogenesis in the NMR. The testicular structure as well as spermatogenic cell types and its organization in breeding, subordinate and disperser males were studied using light microscopy and transmission electron microscopy. Even though the basic testicular design in NMRs is similar to most Afrotheria as well as some rodents with intra-abdominal testes, the Sertoli and spermatogenic cells have many atypical mammalian features. Seminiferous tubules are distended and contain large volumes of fluid while interstitial tissue cover about 50% of the testicular surface area and is mainly composed of Leydig cells. The Sertoli cell cytoplasm contains an extensive network of membranes and a variety of fluid-containing vesicles. Furthermore, Sertoli cells form numerous phagosomes that often appear as extensive accumulations of myelin. Another unusual feature of mature NMR Sertoli cells is mitotic division. While the main types of spermatogonia and spermatocytes are clearly identifiable, these cells are poorly organized and many spermatids without typical intercellular bridges are present. Spermatid heads appear to be malformed with disorganized chromatin, nuclear fragmentation and an ill-defined acrosome formed from star-like Golgi bodies. Rudimentary manchette development corresponds with the occurrence of abnormal sperm morphology. We also hypothesize that NMR testicular organization and spermiation are modified to produce spermatozoa on demand in a short period of time and subsequently use a Sertoli cell “pump” to flush the spermatozoa into short tubuli recti and simplified rete testis. Despite the difficulty in finding cellular associations during spermatogenesis, six spermatogenic stages could be described in the NMR. These numerous atypical and often simplified features of the NMR further supports the notion of degenerative orthogenesis that was selected for due to the absence of sperm competition.

## Introduction

Naked mole-rats (NMRs, *Heterocephalus glaber)* represent one of only two eusocial mammals (Jarvis, 1981, 1985; Faulkes et al., 1991; O’Riain et al., 2000) with reproduction typically restricted to a single female (queen) and 1–3 males within large colonies varying from 40 to 90 individuals. Multiple-paternity has been recorded for this species (Faulkes et al., 1997), but usually the queen selects a male for life and most other males and all other females (subordinates) are reproductively suppressed (Faulkes and Abbott, 1991; Faulkes et al., 1991). This restriction of breeding to a small subset of the population presents a low risk for sperm competition and it is assumed to have shaped the sperm structure (simple and low percentage of normal forms) and motility (slow) in this species (van der Horst et al., 2011). It is not evident at what point during spermatogenesis a path is followed which produces the remarkable heterogeneity of morphologically abnormal spermatozoa in the NMR.

Spermatogenesis is a complex but highly coordinated process which is regulated by paracrine, autocrine, juxtacrine, and endocrine pathways (Chocu et al., 2012). Mammalian spermatogenesis is divided into three phases: mitotic divisions of the spermatogonia and differentiation into spermatocytes (spermatocytogenesis); two consecutive meiotic divisions of the spermatocytes to produce spermatids; and differentiation of the spermatids into spermatozoa (spermiogenesis). During spermiogenesis, structural changes occurring in several cellular compartments include repackaging of the chromatin for transport, development of the acrosome, elongating of the tail, formation of the mitochondrial sheath in the midpiece and reduction of cytoplasmic volume. Here after spermatozoa are released into the seminiferous tubule lumen during the process of spermiation. Seasonality, reproductive lifespan, mode of fertilization and sperm competition are all factors that could influence the organization of spermatogenesis within the testis (Ramm et al., 2014).

Although several papers have appeared on spermatogenesis in the NMR since the early 1990s (Faulkes et al., 1991; Yang et al., 2017), these papers covered a general description of spermatogenesis using wax embedding and hematoxylin-eosin stained sections for brightfield microscopy. The emphasis of these studies was to discern the incidence of spermatogenesis, particularly in relation to the presence/absence of spermatozoa. Their general consensus was that spermatogenesis occurs in breeders and non-breeders, particularly if they were kept separate from the queen, providing evidence of her suppressive role in relation to spermatogenesis specifically.

The first study to pursue greater detail of these processes using transmission electron microscopy was by Onyango et al. (1993) focusing exclusively on non-breeders. Despite this shortcoming, they described most of the cell types of spermatogenesis, albeit without any cellular associations, and related their findings to other mammals. While only few late spermatids or spermatozoa were observed in their investigation, van der Horst et al. (2011) and van der Horst and Maree (2014) described the vastly abnormal spermatozoa in NMR breeders, subordinates and dispersers using both stained sperm smears and electron microscopy. The latter two studies concluded that NMR spermatozoa represent a case of degenerative orthogenesis and it was proposed that this was selected for in the absence of sperm competition.

The aim of the current study is to quantify the testicular structure in the NMR, with special reference to spermatogenesis. We hypothesize that due to the lack of sperm competition there will be degenerate testicular features and “relaxed” spermatogenesis. We investigated testicular ultrastructure and spermatogenesis among breeders, subordinates and dispersers and compared our results to other mammals.

## Materials and Methods

### Animal Husbandry

The study population originated from various localities in Kenya, and was comprised of 10 colonies housed in separate artificial burrow systems at the University of Cape Town, South Africa. Husbandry details have been described previously by Jarvis (1991). A total of 26 male NMRs (*Heterocephalus glaber*) were used in this study, including 10 breeders and 10 subordinates from 10 colonies and 6 males that had been isolated from their colonies for a minimum of 2 months. Two of these isolated males had acquired the dispersing male phenotypes (O’Riain et al., 1996). Breeders were adult males confirmed to be in consort with the queen (mutual naso-anal grooming and copulation). Subordinate males were adult males who did not engage in either naso-anal grooming or copulations with their queens. It should be noted that the “subordinate” NMR males referred to in this study is of the same reproductive status as that of “non-breeding” males used in previous NMR studies.

Ethical clearance for the study was obtained from both the University of Cape Town (2005/V7/JOR), the University of the Western Cape (ScR1RC2007/3/30) and Stellenbosch University (P07/09/019). Animals were removed from their custom made burrow systems and anesthetized with halothane by putting a mask over the head. Surgical anesthesia was attained within 5 min and longer exposure to halothane was used to euthanize animals. The entire reproductive system was dissected out and put into Ham’s F10 medium (Invitrogen, Cape Town, South Africa) at 28°C (NMR body temperature) (van der Horst et al., 2011).

Immediately upon removal of the reproductive systems, small sections of representative parts of the testes were fixed in 2.5% phosphate buffered glutaraldehyde (4°C) and were subsequently routinely processed for transmission electron microscopy (TEM). During TEM processing, 1 μm epoxy sections were cut for viewing and final trimming using an ultra-microtome. The 1 μm epoxy sections have been used to great effect in this investigation to provide high quality brightfield light microscopy images at low magnifications for measurement of different parts of the testes (see later). The ultrathin sections have been used for TEM.

### Capturing of Images for Light Microscopy, Measurement, and TEM

For brightfield microscopy, a Nikon E50i microscope was used and images were captured using an Ace ACA 1300-200uc Basler camera and the Morphology Module of the Sperm Class Analyzer (SCA, version 6.4.0.64) computer-aided sperm analysis software (Microptic S.L., Barcelona, Spain). The sections investigated for this purpose represented 1 μm epoxy sections stained with toluidine blue. The measurement tool of the SCA system was used to measure the seminiferous tubule diameters and to calculate surface areas for both the interstitial tissue and the seminiferous tubules. For TEM, a Jeol JEM 1011 transmission electron microscope (Advanced Laboratory Solutions, Johannesburg, South Africa) at 80 kV was used to capture micrographs of different testicular cells and compartments for subsequent description. More than 1000 micrographs from all 26 males were studied for description of spermatogenic cell types, cellular associations, and staging (see below).

### Statistical Analysis

MedCalc, Version 14 (Mariakerke, Belgium) was used for statistical analyses. Descriptive statistics was used for calculation of averages and standard deviations (SD). Comparisons of testicular morphometry parameters were performed using either ANOVA or Kruskal–Wallis among the different groups and *P* < 0.05 was considered significant.

## Results

### Testicular Compartments

**FIGURE 1:**
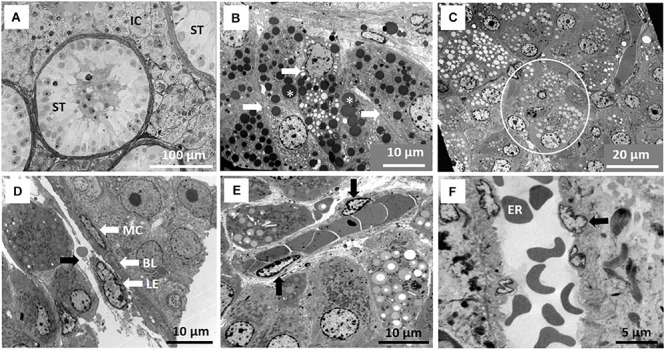
Testicular compartments of the naked mole-rat (NMR). **(A)** Seminiferous tubules (ST) and large interstitial compartment (IC) of the testis. **(B)** Detail of Leydig cells (arrows) arranged in groups of three to four in islands. Lipid droplets (asterisk) are contrasted by osmium tetroxide, appearing black. **(C)** Interstitial compartment containing numerous Leydig cell islands (circle) with lipid content possibly washed out of vesicles during processing. **(D)** Wall of the seminiferous tubules, the lamina propria, consisting of three layers, namely a myoid epithelium (MC), a lymphoid epithelium (LE) and a basal lamina (BL) and arrow indicating lymphatic space. **(E)** Oblique section of arteriole in close association with Leydig cell islands. **(F)** Enhanced view of arteriole in longitudinal section showing endothelial cells (arrow) and erythrocytes (ER).

The interstitial component of the testis in the NMR is large and composed mainly of Leydig cells, blood vessels and lymphatics, representing about 50% of the testicular surface area (Figure 1A). Analyses of breeders, subordinates and dispersers of the same body weight and stage of testicular development show that seminiferous tubules occupy about 45 to 55% of the testicular surface area. In NMRs the average diameter of the seminiferous tubules varies from 152 to 240 μm and there are no significant differences (*P* = 0.18) among the three male groups (Table 1).

**TABLE 1:** Comparison of seminiferous tubule diameter among three groups of naked mole-rat males (*n* = 3 for each group and *n* = 9 in total).

	**Average (μm)**	**±SD (μm)**	**Range (μm)**
Breeders	192.3	20.5	158–240
Subordinates	200.1	18.7	170–234
Dispersers	184.8	20.4	152–219

*At least 10 seminiferous tubules were measured per group (100 seminiferous tubules in total).*

The Leydig cells are arranged in groups (islands) of three or more cells per island and each island is contained within a layer(s) of loose connective tissue (Figures 1B,C). The Leydig cells have a spherical or slightly oval nucleus and contain mainly euchromatin (genetically active). The cytoplasm of the Leydig cell contains numerous mitochondria as well as vesicles with apparently two types of lipid. One type of vesicle appears transluscent/light or containing no lipid or lipid in a particular form. In the second type of vesicle, the lipid appears dark/blue at light microsope level and as electron dense (black) in TEM. These lipid vesicles originate from an extensive network of smooth endoplasmic reticulum. The lymphatic component is clearly defined as sinusoids containing lymph (Figure 1D, black arrow). Arterioles are evident (Figure 1E) in the interstitial compartment with three to four endothelial cells observed in transverse sections. These blood vessels appear in close association with several islands of interstitial tissue (Figure 1E). Figure 1F provides an enlarged view of the endothelial wall of an arteriole.

The wall (lamina propria) of the seminiferous tubules consists of three typical layers, namely an inside basal lamina, a myoid epithelial layer and an outer lymphatic endothelium (Figure 1D). A detailed description of all the cells contained within the seminiferous tubules (spermatogenic and non-spermatogenic), as well as their cellular associations and accordingly staging of spermatogenesis follows below. These cellular features and organization were noticed in males from all three reproductive groups.

### Cell Types of the Seminiferous Epithelium

**FIGURE 2:**
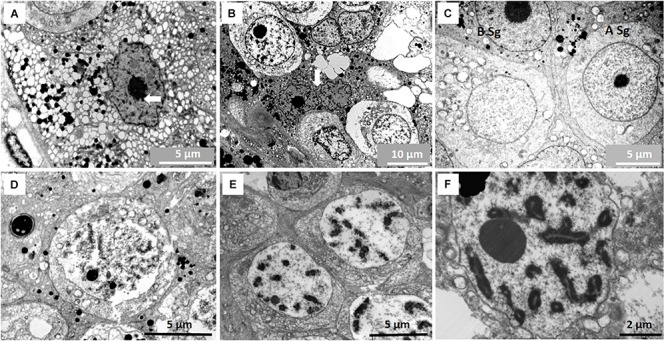
**(A)** Sertoli cell with vast number of fluid-filled vesicles in its cytoplasm. Nucleolus of Sertoli cell is surrounded by peri-nucleolar chromatin (arrow). **(B)** Dividing Sertoli cells (arrow) between two spermatogonia. Similar dividing Sertoli cells are common in all males studied. **(C)** A type spermatogonium (A Sg) and B type spermatogonium (B Sg). **(D)**Leptotene primary spermatocyte. **(E)** Early pachytene primary spematocyte. **(F)** Mid-pachytene primary spermatocyte showing paired chromosomes.

Sertoli cells in the NMR contain many structural differences compared to other mammals. Their nuclei are usually irregular to ovoid with a prominent nucleolus surrounded by a ring of peri-nucleolar chromatin. In almost all testicular sections of most NMR males, we observed Sertoli cells in division and these cells have an extensive cytoplasm filled with numerous membrane-limited vesicles apparently containing fluid (Figures 2A,B). Smaller vesicles seem to coalesce to form large fluid-filled vesicles that might be important in the release of spermatids (see below). The Sertoli cell cytoplasm also often contains many other peculiarities including phagosomes that are described in a separate section below.

**FIGURE 3:**
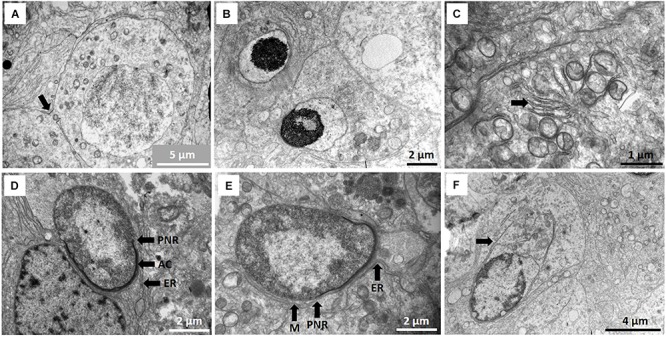
**(A)** Pre-leptotene primary spermatocyte showing inter-cellular bridge (arrow). **(B)** Early round spermatids showing some nuclear condensation just prior to the cap phase of acrosome formation. **(C)** Star-like Golgi figures (arrow) as part of acrosome formation. Location of Golgi apparatus in Sertoli cell indicated with asterisk in Figure 4D. **(D)** Cap phase of spermiogenesis showing acrosome formation (AC) and associated endoplasmic reticulum (ER) as well as the peri-nuclear ring (PNR). **(E)** Start of manchette (M) formation behind peri-nuclear ring (PNR). Endoplasmic reticulum (ER) is associated with the acrosome. **(F)** Caudal tube (arrow) as end phase of manchette.

Dark and light, large A type spermatogonia as well as B type spermatogonia with prominent nucleoli could be identified (Figure 2C). All primary spermatocyte types (pre-leptotene, leptotene, zygotene and pachytene) are present in the NMR testis (Figures 2D–F). Dividing primary spermatocytes are clearly designated and some contain intercellular bridges (Figure 3A). However, subsequent divisions of these cells do not appear to have intercellular bridges (see later on number of spermatids released).

The four main developmental phases of spermatids could be designated in the NMR. Early round spermatids (Golgi phase, Figure 3B; also see later Figures 5A,B for description of spermatid types) are often associated with a large, star-like arrangement of the Golgi apparatus (Figure 3C) that eventually appear to contribute to construction of the acrosome. Round to oval spermatids are showing acrosome formation (cap phase, Figure 3D; also see later Figure 5B) and the peri-nucleolar ring is also formed. Many microtubules are associated with the Golgi phase and also associate with the acrosome.

**FIGURE 4:**
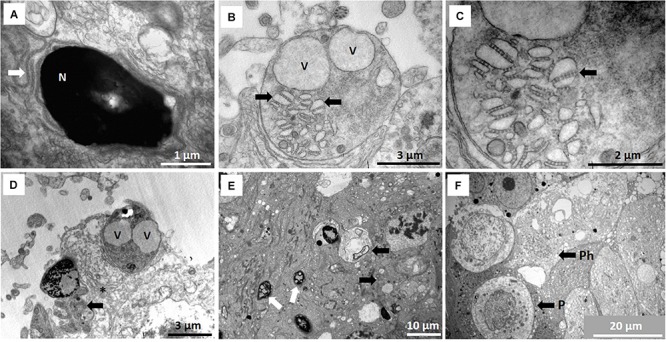
**(A)** Redundant microtubules presumably of manchette origin forming snake-like arrangement (arrow) posterior to spermatid nucleus (N). **(B)** Microtubules form associations with vesicles (arrows) and seem to be involved in spermiation and vacuoles (V) represent crypts after release of late spermatids/spermatozoa. **(C)** Larger magnification of **(B)** showing the microtubules (arrow) in groups of four to eight apparently involved in movement of vesicles to combine and assist in final stage of spermiation. **(D)** Typical cytoplasmic droplet components are shed before sperm release in seminiferous tubule lumen. Arrow indicates part of process where redundant cytoplasm is eliminated before sperm release from vesicle (V). Asterisk indicates center of a remnant star-like Golgi body. **(E)** Different morphological forms of spermatid heads already showing fragmentation (white arrows) as part of spermiogenesis and phagosomes (black arrows). **(F)** Unusual, typical phagocyte (P) in seminiferous epithelium and phagosomes inside Sertoli cell (Ph).

**FIGURE 5:**
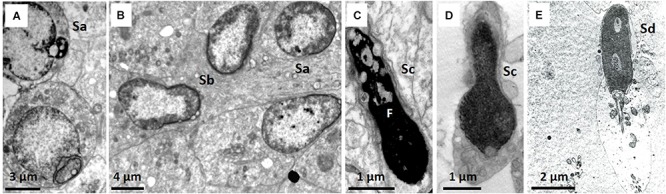
**(A–E)** Morphological details of the four developmental stages of the spermatids. **(A)** Sa, round spermatid also showing acrosomal granule and part of Golgi phase. **(B)** Sb, spermatid with acrosome formation and slight elongation. **(C)** Sc, elongated spermatid showing fragmentation (F). **(D)** Sc, lobe-shaped elongated spermatid. **(E)** Sd, elongated mature spermatid/sperm.

During the phase of spermatid elongation, a simple/rudimentary manchette of microtubules is formed below the perinuclear ring (Figure 3E). This structure is first observed at the acrosome formation stage as a single row of microtubules and in longitudinal sections it appears as a chain of connected microtubules. Several cisternae of endoplasmic reticulum also appear at this stage in the Sertoli cell cytoplasm (usually associated with cell adhesions) and are arranged in the area of the spermatid acrosomal cap (Figures 3D,E). In late spermatids with elongated heads, a clear caudal tube (posterior segment of manchette) emerges (Figure 3F) at the posterior part of the head and seems to unwind in a snake-like fashion (Figure 4A). Elongated spermatids are also depicted in Figures 5C,D in the description of spermatid types. It furthermore seems that microtubules at the posterior part of the sperm head break up into small groups of six to eight microtubules and associate with smaller Sertoli cell vesicles (Figures 4B,C).

Finally, the release of mature spermatids/spermatozoa from the Sertoli cells appear to be assisted by this microtubule mechanism of forming larger vesicles and accompanied by vast Sertoli cell fluid formation (Figures 2A, 4B–D). Figures 4B–D typically show residual bodies with two vacuoles presumably form which two spermatids that have been released. We refer to this “excessive” fluid formation (compared to any other mammalian species) as the Sertoli cell pump – a novel adaptation in mammals and unique to the NMR, as will be discussed.

Many spermatid morphological abnormalities are evident during spermiogenesis. The major atypical aspects are the heterogeneity of head morphology and the large number of spermatid and sperm heads showing fragmentation (Figure 4E). Many residual bodies seem to contain the remains of the vesicles that released the spermatozoa, many of which are presumably phagocytosed by the Sertoli cells forming phagosomes (Figures 4E,F). These phagosomes typically exhibit formation of myelin figures which may be partly related to end products of the phagocytosed material (see section below). A further novel and surprising finding in NMR is the presence of phagocytes (unusual) as part of the seminiferous epithelium (Figure 4F).

### Staging of Spermatogenesis

Staging of spermatogenesis has only been performed in sections of seminiferous tubules presenting with most spermatogonia, spermatocytes, spermatids, and spermatozoa and was more distinct in males sacrificed close to estrous of the queen. Spermatogenesis in the NMR appears to be helical, similar to humans, and more than one stage (spermatogenic cellular association) can be designated in a transverse section of the seminiferous tubules. However, defining these cellular associations in NMRs is complicated due to the heterogeneity in spermatid morphology. It was accordingly important to first distinguish the four spermatid developmental stages (Figure 5), namely: Sa, representing round spermatids in Golgi phase (Figures 3B, 5A); Sb, representing acrosome development, peripheral nuclear condensation and start of elongation (Figures 3D,E, 5B); Sc, representing more pronounced elongation and nuclear condensation (Figures 5C,D); and Sd, representing mature elongated/mature spermatids (Figure 5E).

**FIGURE 6:**
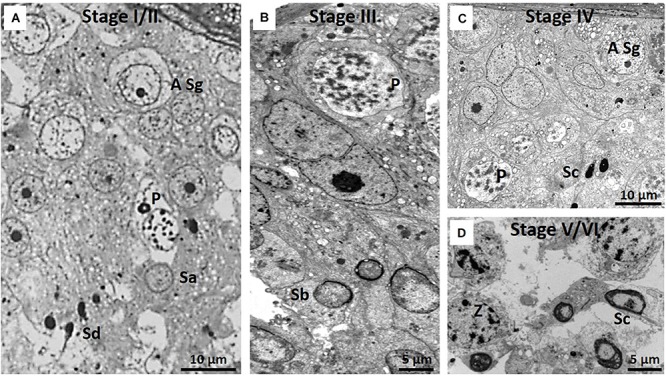
**(A–D)** Six spermatogenic stages of the NMR. Note that Stages I and II and V and VI show many overlapping features and have been grouped together as Stage I/II and Stage V/VI. Since all stages show A type spermatogonia, they have not been indicated in every stage. **(A)** Stage I/II showing A type spermatogonium (A Sg), pachytene primary spermatocyte (P) and spermatid stages Sa and Sd. **(B)** Stage III, pachytene primary spermatocyte (P), Sb spermatids and Sertoli cell (SC). **(C)** Stage IV, A type spermatogonium (A Sg), pachytene primary spermatocyte (P) and Sc spermatid. **(D)** Stage V/VI with zygotene primary spermatocytes (Z) and Sc spermatids. Only zygotene spermatocytes and Sc spermatids are indicated as this combination distinguishes stage V/VI from the other stages.

Despite the limitations posed by spermatid heterogeneity, Figure 6 illustrates six spermatogenic stages and Table 2 lists the different cellular associations of spermatogenesis as shown in these micrographs. Delineation of these six stages was mainly based on an association of spermatogonia, specific primary spermatocytes and the presence of one or two of the four spermatids types. For example, NMR Stage IV contains, as viewed successively from the basal lamina, A type spermatogonia, B type spermatogonia, leptotene and pachytene primary spermatocytes and mainly type Sc spermatids (Figure 6C). For the sake of simplicity and difficulty to consistently distinguish between Stages I and II as well as V and VI, they were respectively grouped together as I/II and V/VI (Table 2).

**TABLE 2:** The cellular associations used in defining the spermatogenic stages of the naked mole-rat.

**Stage**	**Spermatogonia (Sg) and spermatocyte associations**	**Spermatid types**	**Depicted**
			
I/II	A type Sg, Leptotene, Pachytene	Sa (round), Sd (elongated)	Figure 6A
III	A type Sg, Preleptotene, Pachytene	Sb (acrosome formation)	Figure 6B
IV	A type Sg, Leptotene, Pachytene	Sc (condensed/elongated)	Figure 6C
V/VI	A type Sg, Zygotene	Sc, Sd (elongated/mature)	Figure 6D

*Sa, Sb, Sc, Sd = four developmental stages of spermatids.*

### Tubuli Recti and the Rete Testis

**FIGURE 7:**
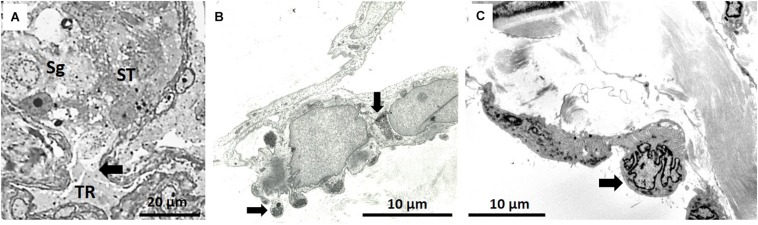
**(A)** Arrow indicates the transition between seminiferous tubule (ST) [with spermatogonium (Sg) indicated] and tubuli recti (TR). **(B)** Typical epithelium of tubuli recti with glycogen stores, as indicated by two arrows. **(C)** Section of rete testis with polymorph nuclear cells (arrow).

The transition between a seminiferous tubule and the tubuli recti is shown in Figure 7A. The tubuli recti in NMRs are short but have the same basic structure as in other mammals. Their cuboidal type cells contain a very large, irregular nucleus and the cytoplasm forms undulations projecting into the lumen that is packed with glycogen granules (Figure 7B). The tubuli recti are confluent with the rete testis which has a large lumen and the cuboidal cells contain short microvilli (Figure 7C).

### Other Features of the Testis

**FIGURE 8:**
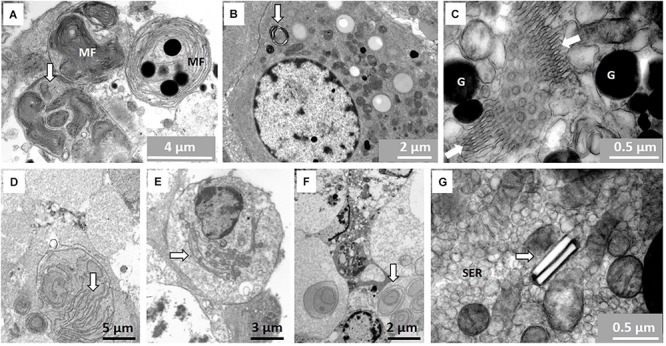
**(A–G)** Features not commonly seen in the testes of mammals. **(A)** Myelin figures (MF) in the form of concentric layers of presumably lipid composition on right and arrow showing more myelin figures with tighter arranged membranes inside a phagosome. **(B)** Typical myelin figure (arrow) in Leydig cell cytoplasm. **(C)** Annulate lamellae (in between arrows) surrounded by granules (G) in Sertoli cell. **(D–F)** Membranes possibly of agranular endoplasmic reticulum (arrows) in residual body and distal part of Sertoli cell close the seminiferous tubule lumen. **(G)** Two presumably para-crystalline structures arranged in groups of two and surrounded by smooth endoplasmic reticulum (SER).

There are numerous structures/organelles in NMRs not commonly observed in the testis of most mammals. Figure 8A shows an example of myelin figures as it occurs in different forms inside the Sertoli cytoplasm and in Leydig cells (Figure 8B). These myelin figures present in Sertoli cells seem to form part of phagosomes. Annulate lamellae within the Sertoli cells are indicated in Figure 8C. These lamellae form many rows of parallel arranged membranes that seem to constrict at specific points and then expand again. There are many examples of these in NMR testes and they have many different types of arrangement. Rows of parallel-oriented and circular membranes are evident, particularly in the distal parts of Sertoli cells and residual bodies (Figures 8D–F). Crystals are present inside the Leydig cells but these do not appear to be a regular feature (Figure 8G).

## Discussion

It has been well-established in the literature that testicular size is larger in breeders compared to non-breeders in the NMR (Faulkes et al., 1991, 1994; Yang et al., 2017). However, in the subsequent discussion we concentrated on the events that occur both in the seminiferous tubules as well as the interstitial cell compartment of breeder, subordinate and disperser NMRs. As we did not find any differences in the cell types among the different groups close to estrous in the queen, the descriptions below will mainly relate to breeders.

### Testicular Compartments and Leydig Cells

The NMR has intra-abdominal testes similar to most Afrotheria (e.g., shrews, moles, aardvark, elephant, manatee, and hyrax), also referred to as testiconds in some rodents (van der Horst, 1972). In many of these testiconds the interstitial tissue is considerably expanded during their breeding season compared to most scrotal mammals (Glover and Sale, 1968; Woodall and Skinner, 1989). In the NMRs used in the present study, the interstitial tissue makes up approximately 50% of the testis surface area/volume, which is similar to the 60% reported for NMRs by Fawcett et al. (1973) and confirmed by others (Onyango et al., 1993; Endo et al., 2002). There is no clear reason for this phenomenon and there are exceptions in testiconds with interstitial tissue only comprising about 9% of testicular volume in some shrews (Kisipan et al., 2014).

The volume of interstitial tissue or the area of the testis occupied by seminiferous tubules seem to be linked to levels of sperm competition in some species, with monogamous species or species with limited male intrasexual competition having a smaller testicular area occupied by tubules (delBarco-Trillo et al., 2013; Peirce et al., 2018). An investigation of various murine rodents by Peirce et al. (2018) revealed that in some, but not all, species with a lower relative testis mass (and thus lower sperm competition level), the seminiferous tubules made up 60–70% of the testis area. While no difference between breeders, subordinates and dispersers were found in the current study in terms of the percentage testis area occupied by seminiferous tubules, Mulugeta et al. (2017) indicated a larger interstitial (Leydig) cell content for NMR breeders compared to non-breeders. In another social mole-rat species, Ansell’s mole-rat (*Fukomys anselli*), the relative area occupied by Leydig cells and seminiferous tubules was also not significantly different between breeders and non-breeders (Montero et al., 2016).

A special feature of NMR testes is that the seminiferous tubules appear to be filled with fluid and resultantly considerably distended. Despite this observation, the diameter of NMR seminiferous tubules is in the same range as reported for other mole-rats, shrews and murine rodents (Kisipan et al., 2014; Montero et al., 2016; Peirce et al., 2018). Peirce et al. (2018) found a significantly greater mean diameter of the seminiferous tubules in species with a higher compared to those with lower relative testis mass in two of the three murine tribes investigated. Although de Bruin et al. (2012) reported a larger diameter of seminiferous tubules in breeding compared to non-breeding Ansell’s mole-rat males, this was not evident in the study by Montero et al. (2016) on the same species or for NMR as reported by Yang et al. (2017) and in the current study.

Another feature that may be unique to NMR is that Leydig cells form islands (three Leydig cells per island) surrounded by regular/loose connective tissue and closely associated with arterioles. We speculate that the two types of vesicles observed in Leydig cells may reflect different stages of steroidogenesis, eventually producing testosterone. The lipid-rich NMR Leydig cell cytoplasm has been reported before (Fawcett et al., 1973; Onyango et al., 1993; Yang et al., 2017), although no mention was made of different types of lipid droplets (translucent vs. dark) encountered. Yang et al. (2017) observed that the Leydig cell area containing lipid droplets, as well as the density of autophagosomes surrounding these droplets, was larger for NMR breeders compared to non-breeders. The latter study linked the lower plasma testosterone levels in non-breeders to a significant decrease in autophagy observed in these males.

### Spermatogenesis and Formation of Abnormal Spermatids/Spermatozoa

Spermatogenesis was evident in all males (breeders, subordinates, and dispersers) included in this study, as was reported in numerous previous studies (Faulkes et al., 1991, 1994; Onyango et al., 1993; Mulugeta et al., 2017; Yang et al., 2017). The morphology of the NMR spermatogenic cells, including spermatogonia and spermatocytes, are remarkably similar to that of other mammalian species. Early spermiogenesis in NMR is indeed characterized by the typical main phases such as Golgi phase and cap phase. However, here the similarities end in relation to other mammalian species, with the tail composition and maturation phases being unusual (van der Horst et al., 2011). One exception in the early spermatid phase (Golgi phase) is the formation of bizarre and excessive star-like Golgi bodies which seem to contribute to acrosome formation. These Golgi bodies presumably provide vesicles that will contribute to acrosome formation and its remnants still remain close to spermiation (Figure 4D). Furthermore, the elongated spermatids (Sc and Sd) are highly heterogeneous in terms of their head morphology and are often fragmented.

Historically, Fawcett et al. (1971) suggested that manchette formation and function is not related to shaping the form or elongation of the sperm head. Subsequently and recently, several authors have convincingly shown, at both electron microscopic and molecular level, that there are two closely associated developments of cytoskeletal elements linked to protein transport and shaping of the spermatid/sperm head (Lehti and Sironen, 2016; Gunes et al., 2018; Wei and Yang, 2018). The first element is mainly actin-bound (related to ectoplasmic specializations of Sertoli cell) and surrounds both the acrosome area and remainder of the sperm head as part of the acrosome–acroplaxome complex. Secondly, a microtubule-based system that originates below the acrosome and is attached to the peri-nuclear ring is involved in manchette formation and indeed important in elongation of the sperm head as well as determining the morphology of the spermatozoon (Wei and Yang, 2018).

It appears that both the acroplaxome and manchette are temporary structures that, apart from their role in nuclear condensation through elongation, provide an important transport system. The manchette region forms an essential part of intra-microtubule transport (IMT) of a multitude of proteins to the basal body/migrated centrioles in relation to formation of the sperm flagellum. It was suggested that many of these IMT proteins feed into an intra-flagellar transport pathway via several motor proteins (Lehti and Sironen, 2016). Once the sperm tail is formed, the manchette disappears after performing its “zipper” action. This action allows for head elongation due to compression of nuclear proteins that become arginine enriched and converted to smaller protamine molecules and subsequently assist in transport of proteins to the tail (Wei and Yang, 2018). The above authors furthermore provided a large body of evidence that when either manchette formation is faulty/absent or some of the multitude of proteins is lacking, it often results in infertility due to absence of motility.

In the NMR, the manchette appears rudimentary comprising either a single or maximal two layers of microtubules in contrast to other mammals where there are typically multiple layers of microtubules forming a substantial sheath around the posterior part of the sperm head (Fawcett et al., 1971). The high degree of sperm head polymorphisms, fragmentation (ill-condensed chromatin) and short tail lacking a fibrous sheath reported here for late spermatids and previously for spermatozoa (van der Horst et al., 2011) are probably a result of this rudimentary/degenerate manchette. It is thus not surprising that the NMR has many abnormal spermatozoa and poor motility since there is a lack or modification of many key functions in completing spermiogenesis (O’Donnell, 2014).

However, NMR breeders with degenerate spermatozoa are highly successful in impregnating the female, despite van der Horst et al. (2011) and van der Horst and Maree (2014) indicating that mature spermatozoa of NMR breeders are grossly abnormal (only 7% of sperm are morphologically normal) and the percentage motility is less than 14%. These authors explained that in the absence of sperm competition, spermatozoa seem to degenerate and sperm quality decreases dramatically but it is still sufficient to fertilize. Several recent studies on other mammalian and bird species similarly related the presence of a high percentage of abnormal spermatozoa to potentially relaxed sperm competition and a monogamous mating system (Breed et al., 2018; Humann-Guilleminot et al., 2018; Peirce et al., 2018). Dorman et al. (2013) indicated that there seems to be co-evolution of the gametes to aid sperm-zona interactions in the Bandicoot rat (*Bandicota indica*), where a low level of sperm competition is also apparent.

Apart from the correlation between poor NMR sperm morphology/motility and degenerate manchette organization and formation, there are other modifications of the NMR manchette that does not exist or has not been described in other species, which appear to be special adaptations in NMR for sperm release. It is well-known that the manchette essentially disappears in mammals toward the end of spermiogenesis and after flagellar formation. All that remains of the mammalian manchette is its posterior part, namely the caudal tube. In the NMR, the single row of microtubules (caudal tube) seems to remain as a snake-like configuration in relation to the posterior part of the sperm head inside the residual body being formed by the Sertoli cell. Subsequently, the microtubules become re-arranged around small Sertoli cell vesicles, combine to form larger vesicles and these seem to assist to release late spermatids/spermatozoa into the testicular lumen via a Sertoli pressure pump mechanism. Although similar large vesicles in the Sertoli cells are visible or have been reported before in both NMR and Ansell’s mole rat, it was referred to as “lesions” or “vacuoles” and its presence was not discussed (Faulkes et al., 1991; Endo et al., 2002; Montero et al., 2016; Yang et al., 2017). Our hypothesis of the pump mechanism shows some similarities to a simplified spermiation process in a species of toad (*Bufo arenarum*) where the endoplasmic reticulum of the Sertoli cell becomes fluid-filled after stimulation by luteinizing hormone and elongated spermatids are pushed out from their crypts in the Sertoli cell (Burgos and Vitale-Calpe, 1967a, b). In the NMR, typically two mature spermatids/sperm are jointly released into the lumen as is evident from the now empty vacuoles of the residual body (Figure 4). Spermatozoa are usually released in large groups with still intact intercellular bridges as evident in human (Dym, 1977) and other mammals (Hess and Renato de Franca, 2008). This further supports our notion that there are no intercellular bridges in between spermatids close to being released.

Another NMR sperm feature that has not been observed in other mammals relates to the elimination of the spermatid cytoplasm. In mammals, spermatozoa released from the testis possess a cytoplasmic droplet that is eventually shed in the cauda epididymis or vas deferens. However, NMR spermatozoa released in the seminiferous tubule do not contain a cytoplasmic droplet and it appears that all the redundant organelles have been shed before spermatid/sperm release. This apparent modification in the spermiation process is supported by the fact that Sertoli cells lack typical apical processes that push spermatids into the lumen and stalks of cytoplasm that stay in contact with the spermatids up until final release (Beardsley et al., 2006; O’Donnell, 2014). It is possible that in NMRs an alternative mode of cytoplasmic elimination is used as described for non-mammalian vertebrates (Sprando and Russell, 1988; Russel, 1993) and represents yet another feature potentially related to degenerative orthogenesis and the unique NMR spermiation process.

While the above discussion represents a totally new explanation of the mechanism for the release of spermatozoa from the Sertoli cell it still poses the question of how does this degenerative system relates to fertility, as in all the examples we studied the breeders were proven fathers? In our previous papers we have shown the relationship between sperm degenerate features and fertility and lack of sperm competition (van der Horst et al., 2011; van der Horst and Maree, 2014). Accordingly, it becomes apparent at what stage of spermatogenesis sperm morphology becomes abnormal. The early phases of spermatogenesis seem to be normal in the NMR but then during acrosome formation (cap phase), the manchette dictates development of normal sperm morphology and here it is simplified/degenerate. These observations are in agreement with findings of Mulugeta et al. (2017) on differentially expressed genes in the NMR gonads between breeders and non-breeders which indicated upregulation of genes in breeders that are mainly expressed in the post-meiotic stages of spermatogenesis. A possible explanation for the lower sperm numbers and sperm motility that have been reported in NMR non-breeders compared to breeders (Faulkes et al., 1991, 1994) stems from additional molecular data reported by Mulugeta et al. (2017) which indicated that genes involved in the meiotic cell cycle, male genitalia development, gamete generation, spermatogenesis, and sperm motility are upregulated in breeders.

### Staging of Spermatogenesis

Deciphering the spermatogenic stages in the NMR was difficult for two reasons. Firstly, it needs to be emphasized that the well-organized arrangement of groups of specific types of primary spermatocytes or spermatids as in most mammals do not exist in NMRs but rather a more diffuse/disorganized arrangement of these cells. Secondly, there are typically many abnormal spermatids/spermatozoa in the NMR and the basis for staging is to relate a particular association of spermatogonia and spermatocytes to a specific spermatid type. The poorly organized arrangement of the various types of spermatogenic cells, also previously reported by Endo et al. (2002) for non-breeders, is possibly due to the fact that intercellular bridges between closely associated cells are scarce.

In the rat there are 19 different types of spermatids, with each of these spermatids having a specific association with spermatogonia and spermatocyte types, resulting in 14 clear spermatogenic stages (I to XIV). In humans there are only four different spermatid types and accordingly only six spermatogenic stages (I to VI) (Dym, 1977). In the NMR there are also four different types of spermatids that, despite their apparent abnormal and fragmented structure, show some resemblance to those in humans. The spermatid heterogeneity thus posed a challenge to find a coherent system relating to specific spermatid types in defining a specific spermatogenic stage in the NMR. Despite this it was possible to recognize six stages in the NMR that show similarities to human Stages I, II, III, IV, V, and VI. However, in our classification system as indicated in Table 2 many similarities were evident between Stages I and II as well as V and VI and these stages were respectively grouped together. It may accordingly be then conceived that only four stages are evident in the NMR but due to their closer relationship to human staging (as compared to rodents) we prefer using the human classification rather than delineating a new one for the NMR. We have previously pointed out that in humans whom are largely monogamous there is a low risk of sperm competition (van der Horst and Maree, 2014). Accordingly, it is not surprising to see some similarities between NMR and humans in staging of spermatogenesis. Moreover, complexity in the process of spermatogenesis is in parallel with the evolution of mammals. It appears that the efficiency of spermatogenesis decreased with humans, being less efficient than most mammals such as bulls or rats (Thompson et al., 2018).

While it is accepted that the queen suppresses many aspects of reproduction in the colony, does that mean she suppresses spermatogenesis in all the males all the time? Faulkes et al. (1991) found that while there are differences in testicular weight and size between breeders and non-breeders, all males exhibited spermatogenesis. Our findings agree with Faulkes et al. (1991) and in addition we found that when spermatogenesis is studied at the same period of the estrous cycle in the same colony, breeders, subordinates and dispersers will exhibit similar testicular histology and all may produce and release spermatozoa from the testes. Accordingly, when breeders do not contain spermatozoa in the testis, a non-breeder from the same colony will also not have spermatozoa at that point in time. This observation raises another question: are breeding males also suppressed and do not produce spermatozoa at a certain time in the colony? We have investigated this potential phenomenon which will be deliberated in a separate paper in the near future.

### Other Features of the NMR Testis

Myelin figures is considered to be non-specific, concentrically layered, osmiophilic and possibly derived from agranular reticulum. In both the Leydig cells as well as the Sertoli cells there appear areas rich in agranular vesicles and in some parts of the cell they may actually form the concentric layers we observed in these cells in NMR (Neaves, 1973). There is good evidence to suggest that these myelin figures may act as important cholesterol storage centers but are also involved in synthesis of various types of steroids such as testosterone and progesterone (Christensen and Fawcett, 1966). Morita et al. (1999) showed that the myelin figures in testis labeled positively for a family of claudine as in myelinated nerves and are very important in formation of tight junctions. It is accordingly not surprising to find that highly concentrated myelin figures as found in NMR Sertoli and Leydig cells are potentially functional in the testis.

Annulate lamellae within the NMR Sertoli cells form different types of arrangements, one of which rows of parallel-arranged membranes appear to constrict at specific points and then expand again just like the outer nuclear membrane. The bulk of information according to older literature (Wischnitzer, 1970) suggests that these organelles originate from the outer nuclear membrane and are important in messaging between nucleus and cytoplasm. Merisko (1989) attributed that, apart from ATP production and membrane biogenesis, there is macro-molecular trafficking via the pores and regulator of genomic expression. They furthermore seem to associate in many instances with ribosomes and become an active source of protein synthesis. The lamellae in this form can either join rough endoplasmic reticulum or be involved in the formation of endoplasmic reticulum. The most important explanation relating to NMR Sertoli cells is that the vast amount of fluid containing vesicles and membranes as well as the extensive agranular reticulum in Leydig cells may actually also be involved in the formation of annulate lamellae and myelin figures. While we can only speculate, it appears that the rows of parallel or circular membranes in the distal Sertoli cells and residual bodies may be associated with the formation of myelin figures and/or annulate lamellae. Christensen and Fawcett (1966) suggested that these membranes can largely be considered smooth endoplasmic reticulum and accordingly have steroid producing functions as suggested above for myelin figures.

Crystals of Reinke are commonly found in Leydig cells as well as the crystalloid of Charcot-Bottcher in Sertoli cells (Dym, 1977). However, we have found a simple crystal-like structure usually occurring in pairs of two in the Leydig cells of the NMR. These are not products of precipitation of processing material as they are clearly embedded in the cytoplasm. There appear to be many types of so called para-crystalline structures both in Leydig cells as well as in the cytoplasm of Sertoli cells (Sohval et al., 1973). Some are in fact almost tubular like in NMR but none bear a close resemblance. Despite all the speculation about the crystalline structures in Leydig cells there seem to be some association with age. These structures appear in the testes of older men as well as in older animals and do not seem to have a specific function (Sohval et al., 1973).

## Conclusion

This study used ultrastructural features of the testis to describe both interstitial and testicular components and their associated cell types as well as staging of spermatogenesis in the NMR for the first time. Our results suggest that the main differences in testicular structure and spermiogenesis in NMR compared to other mammals pertains to low levels of sperm competition and supports prior investigations on NMR spermatozoa and investigations on birds and rodents (van der Horst et al., 2011; van der Horst and Maree, 2014). This hypothesis also assists to explain the phenomenon of mature abnormal spermatozoa in NMR being a function of testicular development/modification, specifically during spermiogenesis.

The testicular compartments of the NMR revealed several aspects that are atypical for a mammalian species and unique for this eusocial species. The NMR interstitial tissue, comprising mostly of Leydig cells, occupies approximately 50% of the surface area/volume of the testis, similar to many other Afrotheria. Seminiferous tubules in NMR show all the typical cell types expected during spermatogenesis with some important exceptions. Mitotic divisions of the Sertoli cells are often found and their cytoplasm is packed with fluid-filled vesicles. These vesicles merge and eventually forms part of our hypothesized Sertoli cell pump which, in addition to other adaptations for a simplified spermiation process, assists to release mature spermatids into the seminiferous tubule lumen. Instead of several spermatids being closely associated not more than two spermatids are found in close vicinity to each other and their release is typically associated with copious amounts of fluid being released in the lumen which appears to assist spermatid release and hence support our Sertoli cell pump hypothesis.

The most prominent feature in which NMR spermiogenesis differs from that of other mammals is in the structure and formation of the manchette which is simplified/degenerate in NMR. Since manchette formation involving many rows of microtubules is associated with producing normal shaped spermatids and normal sperm morphology, it is expected that there will be many diverse and abnormal sperm forms in NMR. Furthermore, the “remnants” of the manchette is modified in NMR and seems to be associated with the release of spermatids from the Sertoli cells. Intercellular bridges are present during early phases of spermatogenesis but then seem to “disappear” and are not present in spermatids. The lack of a cytoplasmic droplet during release of sperm into the seminiferous tubule lumen emphasize further modification/simplification compared to other mammals and relate to degenerative orthogenesis.

## Data Availability Statement

All datasets generated for this study are included in the manuscript/supplementary files.

## Ethics Statement

The animal study was reviewed and approved by University of Cape Town (2005/V7/JOR), the University of the Western Cape (ScR1RC2007/3/30), and Stellenbosch University (P07/09/019).

## Author Contributions

GH and LM collated the results and drafted the manuscript. All authors read and approved the final manuscript and designed the study, sampling and analyzed the data.

## Conflict of Interest

The authors declare that the research was conducted in the absence of any commercial or financial relationships that could be construed as a potential conflict of interest.
